# Selection of Processing Methods and Parameters for Composite Inserts in Window Profiles with Regard to the Strength of Their Welds

**DOI:** 10.3390/ma18010044

**Published:** 2024-12-26

**Authors:** Marek Kozielczyk, Kinga Mencel, Jakub Kowalczyk, Marta Paczkowska

**Affiliations:** 1 Faculty of Civil and Transport Engineering, Institute of Machines and Motor Vehicles, Poznan University of Technology, 60-965 Poznan, Poland; marek.kozielczyk@doctorate.put.poznan.pl (M.K.); jakub.kowalczyk@put.poznan.pl (J.K.); 2 Faculty of Mechanical Engineering, Institute of Material Technology, Poznan University of Technology, 60-965 Poznan, Poland; kinga.mencel@put.poznan.pl

**Keywords:** poly(vinyl chloride), window profile, weld, breaking load, strength

## Abstract

In the study of structural materials, the analysis of fracture and deformation resistance plays an important role, particularly in materials widely used in the construction industry, such as poly(vinyl chloride) (PVC). PVC is a popular material used, among others, in the manufacture of window profiles, doors, pipes, and many other structural components. The aim of this research was to define the influence of the degree of milling of the glass-fibre-reinforced composite on the strength of the window frame welds, and in the next step, to propose new welding parameters to obtain sufficient strength properties that allow reducing the cost of the technological welding operation. During the tests, it was found that the average failure load of the composite samples was highest at a milling depth of 1 mm and lowest at 6 mm. Up to a depth of 1 mm, the values of destructive loads show an increasing trend, while above this depth, a decreasing trend. A clear reduction in strength was observed when milling to a depth of 1.5 mm, which is related to material discontinuity and the lack of a visible weld joint caused by milling too deep. The differences in average failure loads between the samples of 0 mm, 0.5 mm, and 1 mm milling are minimal.

## 1. Introduction

Key factors affecting the durability and reliability of civil engineering are the quality of the materials and their joints. There is currently a growing trend of PVC production driven by demand and increasing production capacity [1]. In 2019, approximately 6 million tonnes of virgin PVC were produced in the EU. Of this, 5.3 million tonnes were unplasticised PVC and about 0.7 million tonnes were plasticised PVC mixed with other substances. Global PVC production was estimated at 55 million tonnes in 2016 [1]. PVC is an important material used in the production of the window profiles that make up modern civil engineering. One of the key processes used in PVC window technology is the welding process [2,3,4]. The welds should be durable and transfer the required stresses. These properties are ensured by appropriate process parameters, i.e., welding temperature, melting time, melting pressure, clamping pressure, hardening time, and surface quality before welding [5,6,7,8]. A thorough study of the welding process is an opportunity to better understand the possibilities and risks of the process and to make more efficient use of available materials and technologies. It is possible to eliminate steel reinforcements in favour of glass composite reinforcements with a fibre content of 52%. The use of such composite reinforcements as profile filling may lead to a reduction in the weldability of the components to be joined. It can also cause difficulties during the welding process, due to one machine setting temperature and different melting points of PVC and composite [6].

Numerous articles have been published to date on the research of PVC frame profiles in window technology and the welding process. These include the influence of welding parameters on the strength of welded corners using profiles made using the co-extrusion method [6], static calculations of windows under temperature loads [3], and the bending strength of PVC profile welds on welding machines with different numbers of heads [8]. However, these were PVC profiles without reinforcement. It was noted that not only the temperature specified by the PVC profile manufacturer (approx. 264 °C) can provide the minimum required bending strength. Therefore, it is to be expected that the same will be true for other process parameters. Cases have also been described in the literature of a different type of welding than using a 4-head butt welding machine, i.e., ultrasonic welding [9,10,11]. In another case, other materials besides window profiles have also been used in the welding process, i.e., metal mesh as an alternative heating element [12], high-performance polymers for medical purposes [13], or resistance welding, also of glass-fibre-reinforced composites [14,15,16,17]. Friction stir spot welding, which is also used for polymeric materials, has been addressed in the literature [18]. In addition, energy issues relating to PVC profiles themselves have been extensively described in the literature [19,20,21,22,23]. Also, the shape of the profile itself has been considered [24,25], as well as the purpose of the profiles and welded forms, acting as structural elements of the building [26,27].

However, to date, there are no research results in the world literature containing information on the physical and chemical aspects of the tested welded joints of PVC profiles reinforced with glass-fibre composite, in the form of a strength analysis related to the different milling depths of the composite reinforcing the profile. Additionally, there is no analysis of the compatibility of the materials used, the degree of degradation of the material after welding, or the effect of the type of material: virgin versus recycled on the properties of the welded joints. For the most part, the available publications are limited to providing only information on the breaking forces of the joint.

The aim of this research was to define the effect of the degree of milling of the glass-fibre-reinforced composite on the strength of window frame welds and, in the next step, to propose new welding parameters to obtain sufficient strength properties that allow for a reduction in the cost of the technological welding operation.

## 2. Materials and Methods

### 2.1. Research Plan

**Figure 1:**
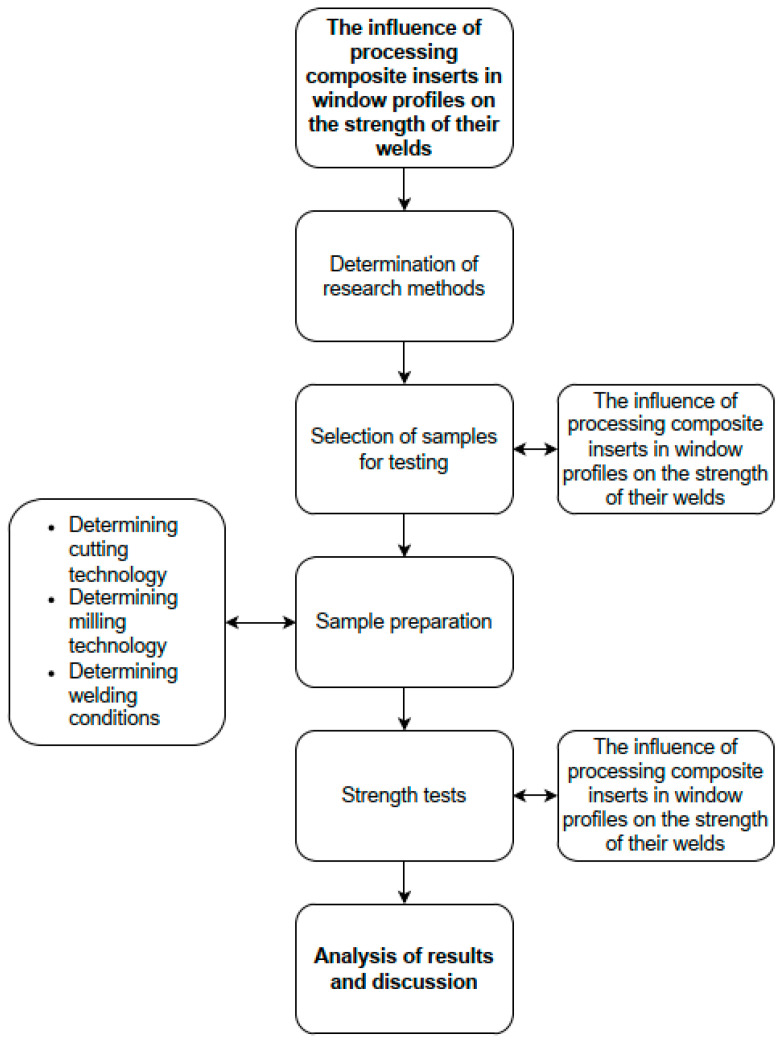
Research plan.

The research was carried out according to the scheme (Figure 1). The main research areas included the selection of samples (window profiles), the determination of their preparation and welding, and strength tests.

For the tests, it was envisaged to use window profiles made of PVC together with a composite insert. It was planned to mill the inserts to different depths, then weld the specimens and evaluate their strength.

### 2.2. Materials and Test Benches

A window profile with an installation depth of 85 mm and a width of 70 mm, of 6-chamber construction, was adopted for the study. This is a popular profile, commonly used for window joinery. The basic properties of the PVC compound are presented in Table 1.

**Figure 2:**
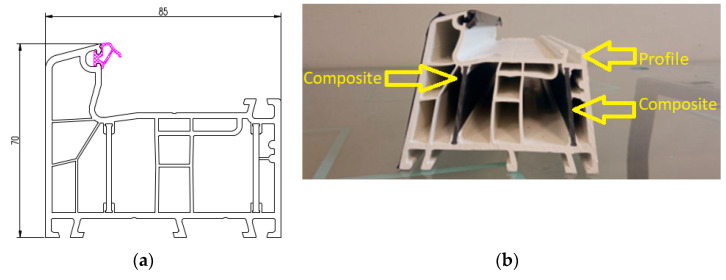
Cross-section of the tested profiles (**a**) and view of the composite inserts (**b**).

A drawing of the profile used is shown in Figure 2a, while Figure 2b shows the area with composite inserts.

Commonly, in order to maintain the appropriate static properties of the building structure, the PVC profiles are reinforced with steel profiles. It is possible to eliminate steel reinforcements in favour of fibreglass composite reinforcements. As a result, there is no need for additional reinforcements. The advantages of using composite reinforcements include very good thermal insulation properties, elimination of thermal bridges, and improved heat transfer coefficient values as a result of eliminating steel reinforcements. Another advantage is the much lighter weight compared to solutions with steel reinforcements.

The profiles to be tested met a number of requirements. Among these was their minimum temperature of 18 °C. This is the minimum value assumed by the profile manufacturer, which allows for the correct processing of the profiles, i.e., cutting or welding, without the risk of defects during the technological process. In addition, the profiles were characterised by an appropriate geometric shape, i.e., they were not bent, twisted, or warped.

In the research, two main stages were adopted. The first was an exploratory study, focusing on carrying out strength tests and obtaining the results of breaking loads for the adopted profile without milling the composite insert. The second direction was to carry out strength tests with prior milling of the composite to specified depths. The milling depths of the composite were further variations in this study. The composite was milled from the largest to the smallest depth: 6.0 mm, 3.0 mm, 2.0 mm, 1.5 mm, 1.0 mm, and 0.5 mm.

For all types of testing (reconnaissance and for subsequent composite milling variants), at least 3 window frames were accepted for testing, which represented 12 individual corner samples, in accordance with the provisions of the standard [4].

**Figure 3:**
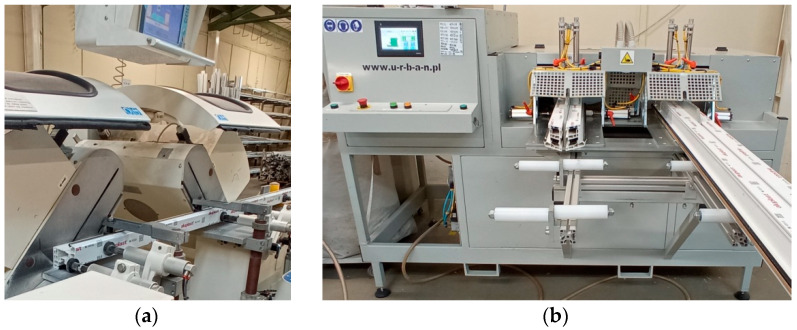
Profile machining; (**a**)—cutting; (**b**)—milling.

In the process of cutting profiles (Figure 3a), an automatic double-head saw DS 150—Gamma manufactured by WEGOMA Polska (WEGOMA Weiss Fensterbau Maschinen GmbH, Bietigheim, Germany) was used. The cutting of profiles was carried out automatically. The machine allowed the accuracy of the required cutting dimension to decimal parts. Before cutting, the heads were calibrated. During cutting, the profile was stabilised by clamps on two axes: vertical and horizontal. The following cutting tool parameters were adopted: disc with carbide blades, disc diameter: 550 mm, disc thickness: 4.2 mm, number of teeth: 120, tooth shape: flat trapezoidal, cutting speed: 3000 rpm, feed: 45 mm/s.

In the process of milling the composite reinforcing the profile, an automatic two-spindle milling machine DFM-202/4 (URBAN Polska Sp. z o.o., Żary, Poland) shown in Figure 3b was used. After arranging the profiles, as shown in the figure, the composite was cut using carbide disc milling cutters. The machine allowed milling to be programmed to one decimal place. In the tests, an 80 mm diameter disc milling cutter with a carbide blade and plate width of 12 mm was used, with a rotational speed of 18000 rpm; the feed rate was 20 mm/s. The profiles in the machine were stabilised with pneumatic clamps in two planes.

**Figure 4:**
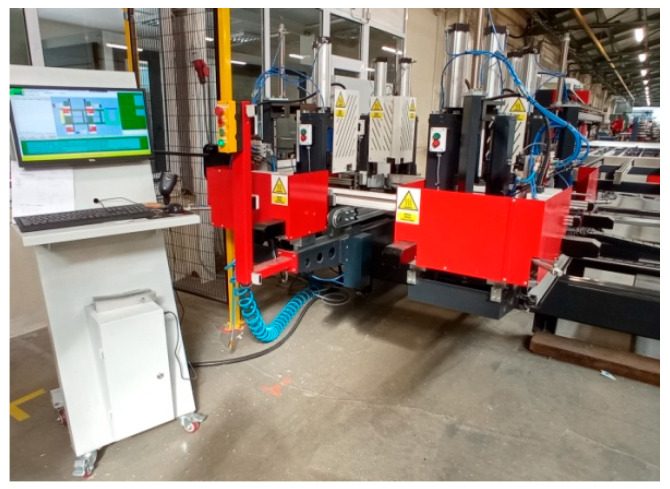
A 4-head welder was used in the tests.

For the welding process, an automatic 4-head welder WSA 4RH/LH manufactured by WEGOMA Polska (WEGOMA Weiss Fensterbau Maschinen GmbH, Bietigheim, Germany) was used (Figure 4). The machine had the ability to enter variables into two decimal places for some parameters. Apart from the manufacturer’s proposed head feed speed, all other parameters had an accuracy of one decimal place. All tests were carried out for a welding temperature of 264 °C, melting time of 30 s, and welding head feed of 0.25 mm/s. To determine the statistics, confidence intervals were adopted assuming a specific number of samples, and a probability of 95%. For the process of cleaning welds from the welding flash, an automatic corner cleaner WPCNC2/4 manufactured by WEGOMA Polska was used.

**Figure 5:**
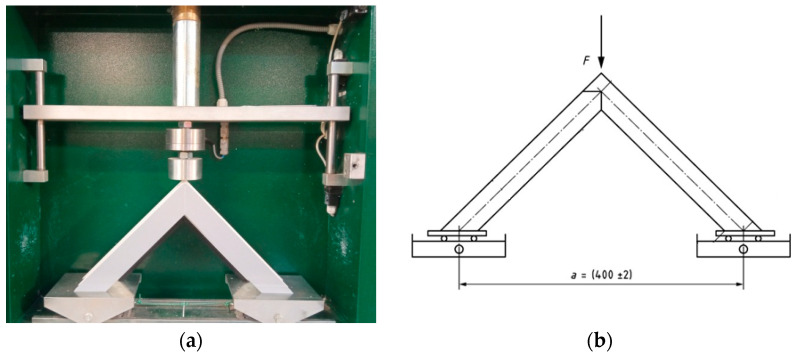
LN2000 corner breaker; (**a**)—sample view; (**b**)—diagram; F—pressure force.

The strength tests used the LN2000 corner breaker (PPHU DELTA, Ksawerów, Poland)—Figure 5. The measurement of the breaking load was performed directly on the breaking punch with a digital readout of the current value. The machine also had a function for storing the maximum value in a given test cycle. The device for testing bending by compression had a load measurement range from 2 kN to 20 kN, the feed speed of the breaking punch was 50 mm/min, and the accuracy of the breaking load measurement was 10 N.

## 3. Analysis of Results and Discussion

### 3.1. Reconnaissance Research

The first case studied (Table 2) contains the results relating to reconnaissance tests to determine the correctness of the welding process and to determine the average failure loads.

The tests prove that there are no larger dimensional deviations from the nominal dimension than 1 mm in the individual axes of the welding machine. Thus, in terms of the expected dimension, the mould is welded correctly.

**Figure 6:**
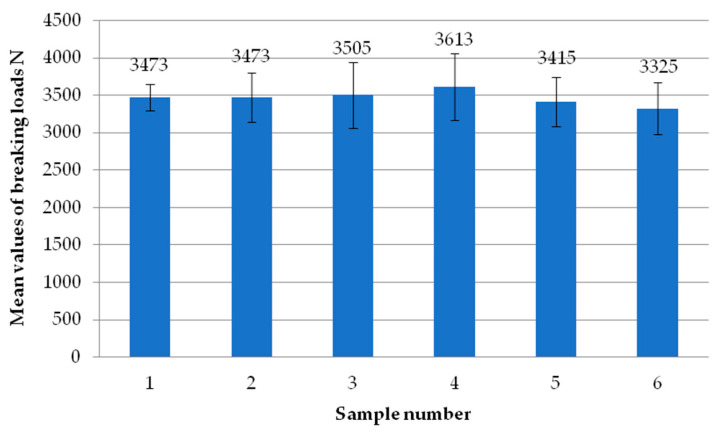
Average failure load values of individual reconnaissance test samples.

Figure 6 shows the average failure loads of the exploratory tests for each sample. Each sample is the average failure load from all welding heads. It can be seen that there are no significant differences in values between the individual tests carried out, suggesting the repeatability of the tests. All samples exceeded the break load threshold of 3000 N, while two of them even exceeded 3500 N. Thus, they significantly exceeded the manufacturer’s indicated limit of 2760 N.

**Figure 7:**
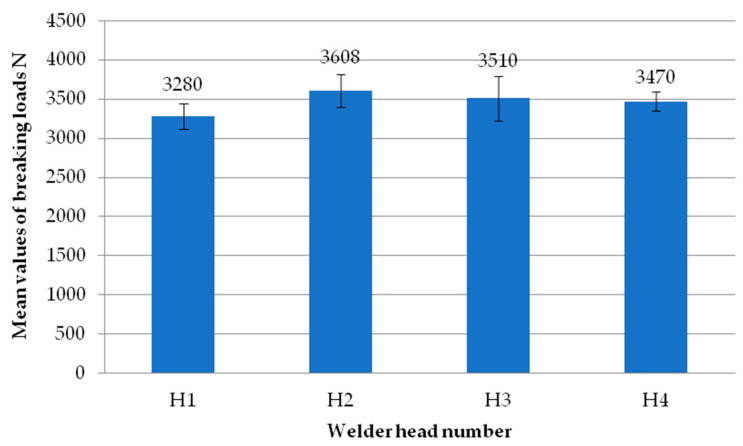
Average values of failure loads for individual reconnaissance test heads.

Figure 7 shows the mean values of the exploratory test failure loads obtained on each of the four heads. Each head represents the average failure load from all the tests performed on that head. It can be seen that there are no significant differences in values between the individual heads. The highest mean value is recorded for the second head. On the other hand, the lowest average value is recorded for the first head. As with the individual samples, each result from the individual heads has an average load exceeding 3000 N.

**Figure 8:**
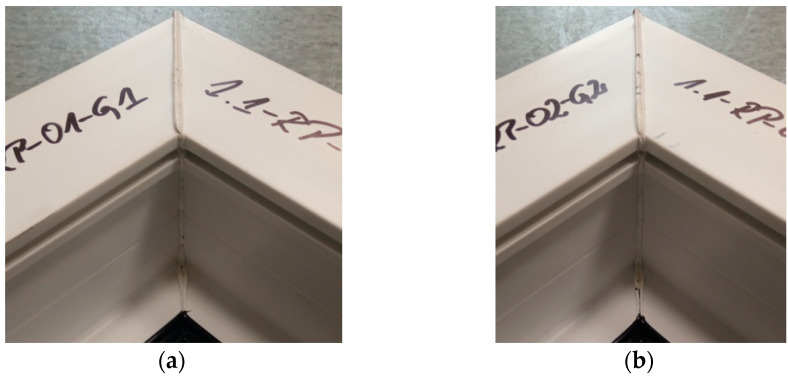
Two sample welds of reconnaissance test samples; (**a**)—for the first head, (**b**)—for the second head.

Figure 8 shows the welds of two different exploratory test samples. All specimens had a white efflorescence colour. This shows that the correct welding temperature was chosen, one that does not degrade the polyvinyl chloride material. All samples retained material continuity within the weld, i.e., a weld seam could be observed in all samples. This is a cast of melted profiles created during the welding process. The symmetry of the welds was also examined. The result was to confirm that there was no displacement of the profiles during the welding process.

### 3.2. Tests for Milling the Composite to a Depth of 6 mm

The second case studied was milling the composite at a depth of 6 mm. Table 3 shows the results of the failure loads from milling the composite to a depth of 6.0 mm.

The analyses showed that the dimensional deviations along the individual axes of the welding machine do not exceed 1 mm from the nominal dimension, which means that the mould is welded as expected.

**Figure 9:**
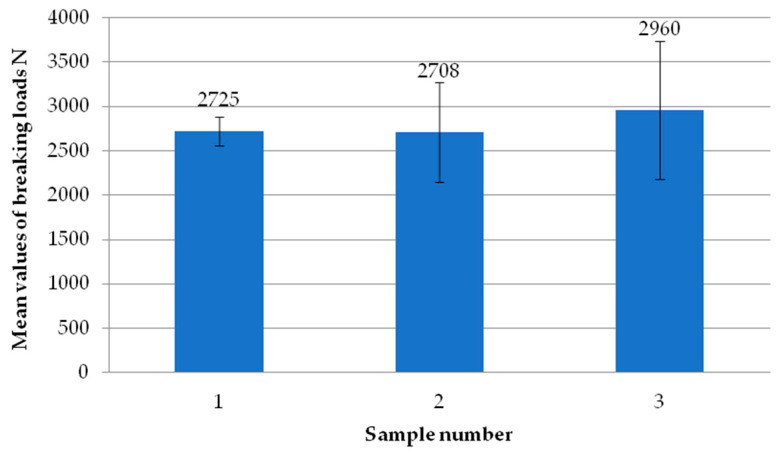
Average failure load values of individual test specimens using milling of the composite to a depth of 6 mm.

Figure 9 shows the average failure load values of the individual specimens using milling of the composite at a depth of 6 mm. No specimen exceeded the average failure load value of 3000 N. In addition, two of the three specimens did not exceed the limit value of 2760 N.

**Figure 10:**
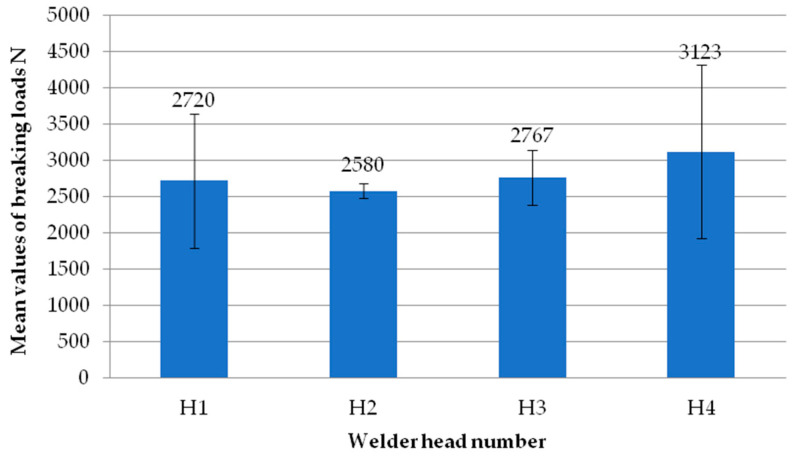
Average failure loads for individual heads using milling of the composite to a depth of 6 mm.

Figure 10 shows the average failure loads for each head. Each head represents the average failure load from all the specimens made on that head using milling of the composite to a depth of 6 mm. It can be seen that there are no significant differences in values between the individual heads. The fourth head has the highest average value. The second head has the lowest average value. Only one, the fourth head, recorded an average breaking load in excess of 3000 N.

**Figure 11:**
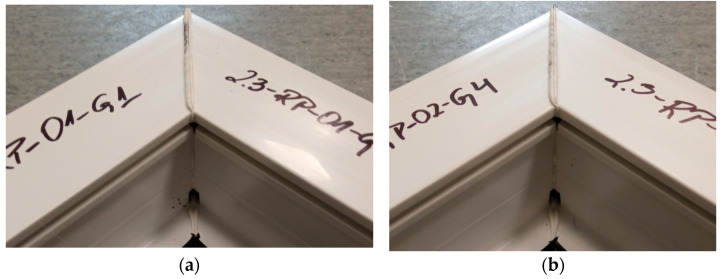
Two selected welds of test specimens using composite milling to a depth of 6 mm; (**a**)—for head one, (**b**)—for head four.

In Figure 11, an example of the welds of two different test specimens can be observed with the composite milled to a depth of 6 mm. All specimens had a white efflorescence colour. This demonstrates that the correct welding temperature was selected. All samples within the welds did not maintain material continuity. Incomplete weld seams could be observed in all samples. This indicates that the composite was milled to too great a depth. The symmetry of the welds was also investigated. The result was confirmation that there was no displacement of the profiles during the welding process. However, the lack of material continuity and spot-missing welds disqualify the sample in the assumed parameters.

### 3.3. Tests for Milling the Composite to a Depth of 3 mm

The third case studied was milling the composite at a depth of 3 mm. Table 4 shows the results of destructive loads using milling of the composite to a depth of 3 mm to determine the correctness of the welding process and to determine the average destructive loads.

The test results confirm that the dimensional differences with respect to the nominal dimension are no greater than 1 mm on each axis of the welding machine, indicating that the mould has been properly welded.

**Figure 12:**
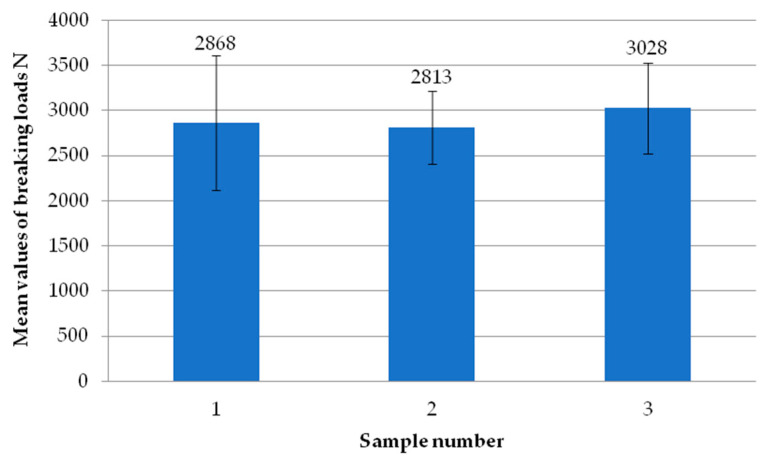
Average failure load values of individual test specimens using milling of the composite to a depth of 3 mm.

Figure 12 shows the average failure load values of the individual specimens using milling of the composite at a depth of 3 mm. It can be seen that there are no significant differences in values between the individual samples. Only one specimen exceeded the mean value of the failure load of 3000 N. The other two, on the other hand, did not exceed an average breaking load of 2900 N.

**Figure 13:**
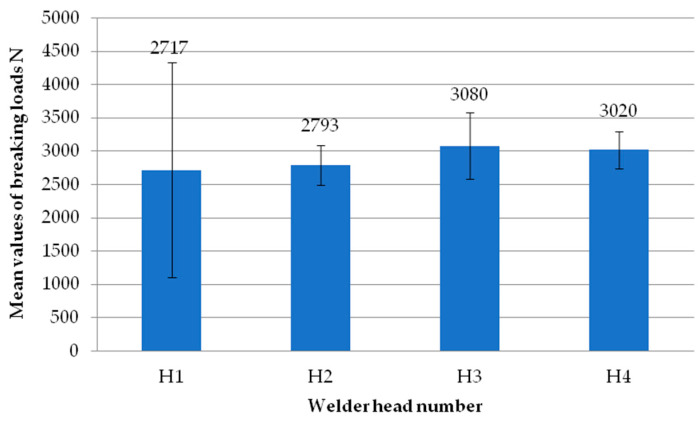
Average failure loads for individual heads using milling of the composite to a depth of 3 mm.

Figure 13 shows the average failure loads for each head. Each head represents the average failure load from all the specimens made on a given head using milling of the composite to a depth of 3 mm. It can be seen that there are no significant differences in values between the individual heads. The third head has the highest average value. The first head has the lowest average value. Only two heads, the third and fourth, recorded average breaking loads in excess of 3000 N. The first head has a greater variation between the results than the other heads. This may be due to the imprecise positioning of the profile in the gearbox. Consequently, inaccurate welding occurred during head feeding.

**Figure 14:**
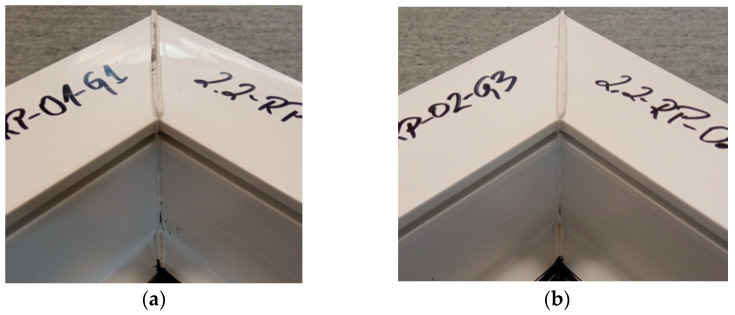
Two selected welds of test specimens using composite milling to a depth of 3 mm; (**a**)—for head one, (**b**)—for head three.

In Figure 14, the welds of two different test specimens can be observed with the composite milling at a depth of 3 mm. All specimens also had a white colour on the flashings. It can be seen from this that an appropriate test temperature was adopted. All samples within the welds did not maintain material continuity. Spot-missing weld seams could be observed in all samples. This indicates that the composite was milled to too great a depth. The symmetry of the welds was also investigated. The result was confirmation that there was no displacement of the profiles during the welding process. However, the lack of material continuity and spot-missing welds disqualify the sample in the assumed parameters.

### 3.4. Tests for Milling the Composite to a Depth of 2 mm

Table 5 shows the results of destructive loads using milling of the composite to a depth of 2 mm to determine the correctness of the welding process and to determine the average destructive loads.

The tests carried out show that the deviations from the nominal dimension do not exceed 1 mm in any of the axes of the welding machine, which makes it possible to conclude that the mould has been correctly welded.

**Figure 15:**
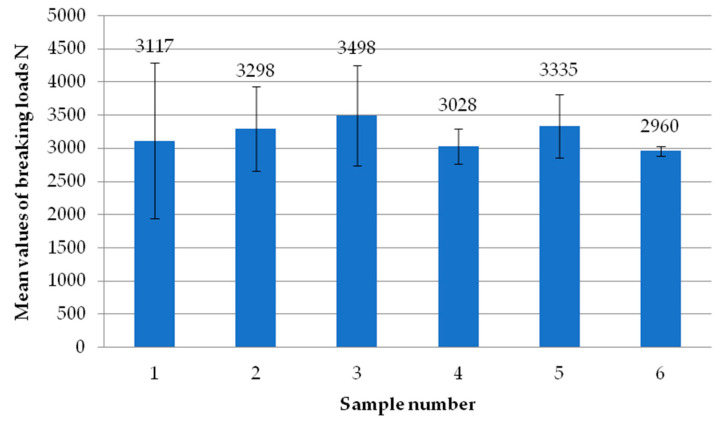
Average failure load values of individual test specimens using milling of the composite to a depth of 2 mm.

Figure 15 shows the average failure load values of the individual specimens using milling of the composite at a depth of 2 mm. It can be seen that there are no significant differences in values between the individual samples. Five of the six specimens achieved results exceeding the breaking load value of 3000 N. During testing, specimen No. 1 from head H2 achieved a result of 1430 N. This was a significant outlier. Therefore, a Dixon test was carried out to investigate whether this result should be rejected. As a result of the calculations, the test confirmed that this single result could be rejected.

**Figure 16:**
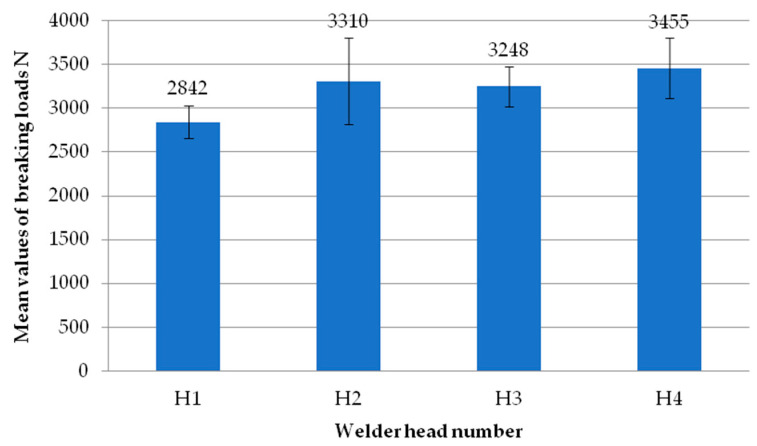
Average failure loads for individual heads using milling of the composite to a depth of 2 mm.

Figure 16 shows the mean values of the failure loads obtained during the testing process for the individual heads. A clear difference can be seen between head one and head four. The mean value of the breaking load for head one was more than 2842 N, while for head four it was 3455 N, and this is the highest value in this comparison. Both heads three and four recorded breaking load values in excess of 3000 N.

**Figure 17:**
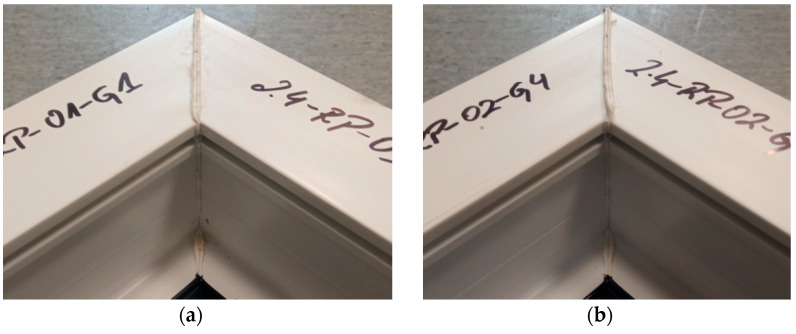
Two selected welds of test specimens using 2 mm deep composite milling; (**a**)—for head one, (**b**)—for head four.

In Figure 17, the welds of two different test specimens can be observed with the composite milled to a depth of 2 mm. All specimens had a white efflorescence colour. This shows that the correct welding temperature was selected—one that does not adversely affect the PVC profile through degradation. All samples within the welds, especially in the corners, did not maintain material continuity. Spot-missing weld seams could be observed in all samples. This indicates that the composite was milled to too great a depth. The symmetry of the welds was also investigated. The result was confirmation that there was no displacement of the profiles during the welding process. However, the lack of material continuity and spot-missing welds disqualify the sample in the assumed parameters.

### 3.5. Tests for Milling the Composite to a Depth of 1.5 mm

Table 6 shows the results of destructive loads using milling of the composite to a depth of 1.5 mm to determine the correctness of the welding process and to determine the average destructive loads.

According to the tests, the dimensions on the axes of the welding machine are within 1 mm of the nominal dimension, indicating that the welding of the mould was carried out correctly.

**Figure 18:**
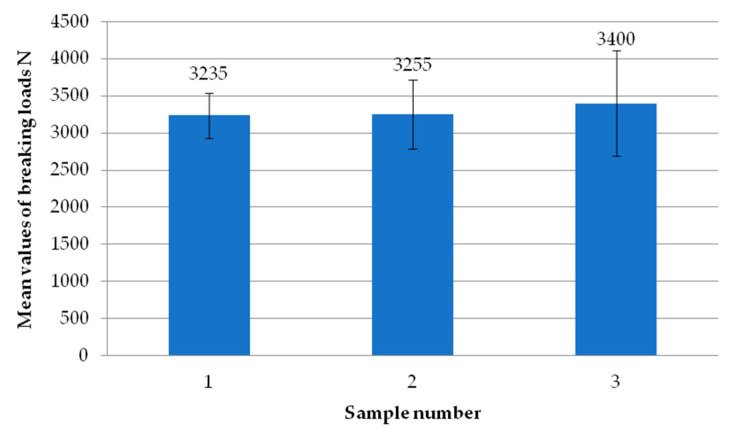
Average failure load values of individual test specimens using milling of the composite to a depth of 1.5 mm.

Figure 18 compares the failure load values for milling the composite to a depth of 1.5 mm. The highest value was achieved by sample three. The other samples obtained values that are not significantly different from the highest value of 3400 N.

**Figure 19:**
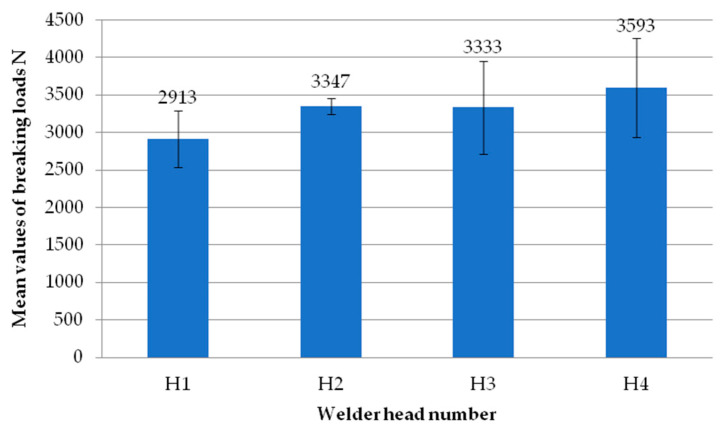
Average failure loads for individual heads using milling of the composite to a depth of 1.5 mm.

Figure 19 shows the average values of the failure loads obtained during the tests for each head. The highest average value is for head four, while the lowest is for head one. Head two, head three, and head four exceeded the value of the breaking loads at 3000 N. The limit value was 2670 N.

**Figure 20:**
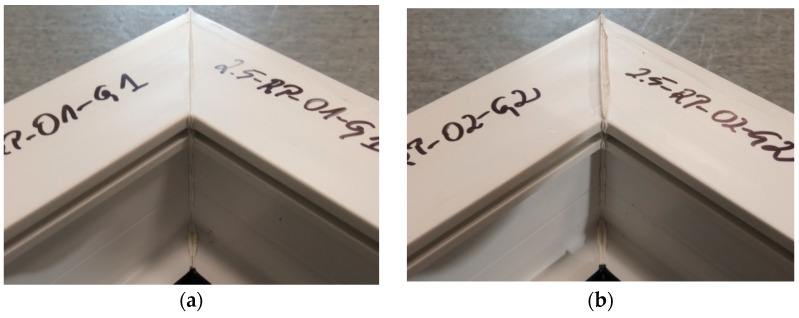
Two different selected welds of test specimens using composite milling to a depth of 1.5 mm; (**a**)—for head one, (**b**)—for head one.

In Figure 20, it is possible to observe the welds of two different test specimens using composite milling to a depth of 1.5 mm. All the specimens obtained had a white efflorescence colour. This shows that the correct welding temperature was selected—one that does not adversely affect the PVC profile through degradation. All samples within the welds, especially in the corners, did not maintain material continuity. In most of the samples, spot-missing weld seams could be observed. This indicates that the composite was milled to too great a depth. The symmetry of the welds was also examined. The result was confirmation that there was no displacement of the profiles during the welding process. However, the lack of material continuity and spot-missing welds in the majority of the specimens disqualifies the specimens in the qualitative context of the assumed parameters.

### 3.6. Tests for Milling the Composite to a Depth of 1 mm

Table 7 shows the results of destructive loading using milling of the composite to a depth of 1 mm to determine the correctness of the welding process and to determine the average destructive loads.

The tests show that the dimensional differences from the nominal values on the axes of the welding machine are less than 1 mm, which means that the mould is correctly welded in the expected dimension.

**Figure 21:**
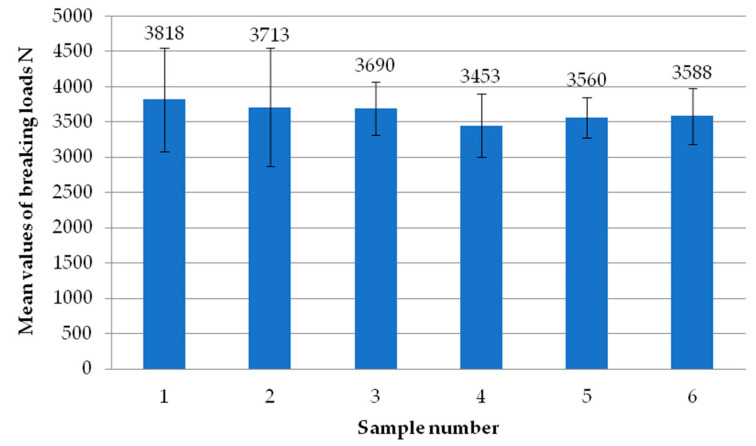
Average failure load values of individual test specimens using milling of the composite to a depth of 1 mm.

Figure 21 compares the failure load values for milling the composite to a depth of 1 mm. All samples exceeded the value of 3000 N. In addition, all but the fourth sample exceeded the value of 3500 N. There were no significant differences in the failure load values for the samples tested in this comparison.

**Figure 22:**
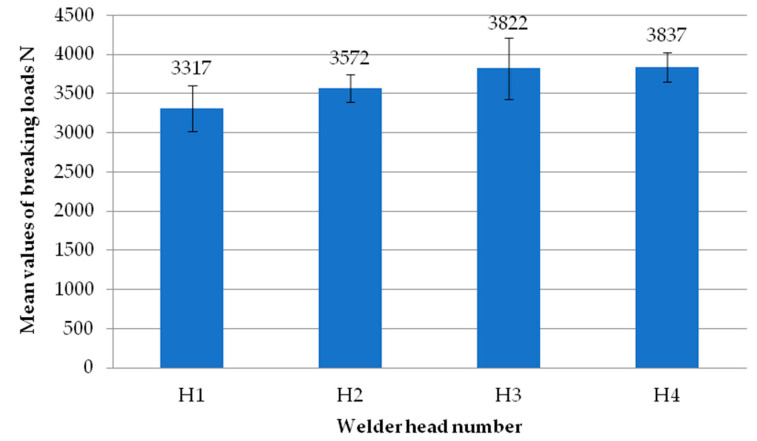
Average failure loads for individual heads using milling of the composite to a depth of 1 mm.

Figure 22 shows the average failure loads for each head. Each head represents the average failure load from all the specimens made on a given head using composite milling to a depth of 1 mm. It can be seen that there are no significant differences in values between the individual heads. The third head has the highest average value. On the other hand, the lowest average value is recorded by head one. As with the individual tests, each of the heads has an average load exceeding 3000 N, while two of the heads exceed 3500 N.

**Figure 23:**
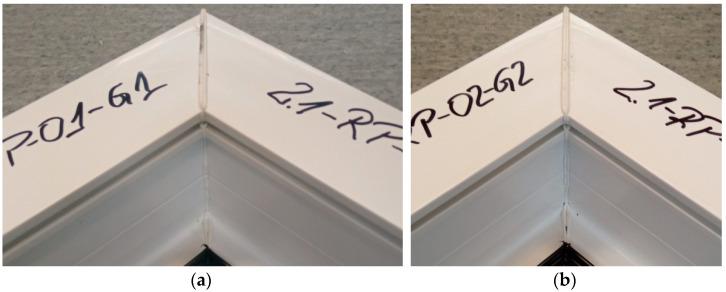
Two selected welds of test specimens using composite milling to a depth of 1 mm; (**a**)—for head one, (**b**)—for head two.

Figure 23 shows examples of the welds of two different test specimens using composite milling to a depth of 1 mm. All the samples had a white efflorescence colour. This demonstrates that the correct welding temperature was selected, which does not adversely affect the PVC profile. In this case, all samples within retained material continuity. The presence of a weld seam could be observed in all samples. The symmetry of the welds was also investigated. The result was confirmation that there was no displacement of the profiles during the welding process.

### 3.7. Tests for Milling the Composite to a Depth of 0.5 mm

Table 8 shows the results of failure loads using milling of the composite to a depth of 0.5 mm to determine the correctness of the welding process and to determine the average failure loads.

The achieved measurement results show that the maximum dimensional deviations from the nominal ones do not exceed 1 mm on the axes of the welding machine and the mould meets the expected dimension assumptions.

**Figure 24:**
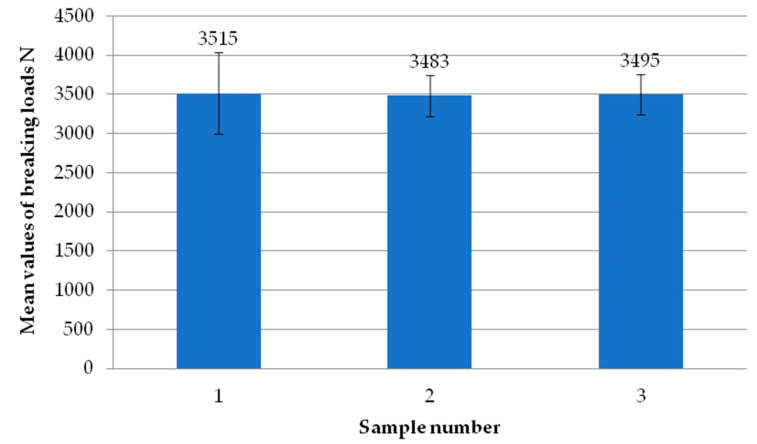
Average failure load values of individual test specimens using milling of the composite to a depth of 0.5 mm.

Figure 24 shows the mean values of the failure loads of the individual specimens using milling of the composite at a depth of 0.5 mm. It can be seen that there are no significant differences in values between the individual samples. All three specimens exceeded the break load value of 3400 N.

**Figure 25:**
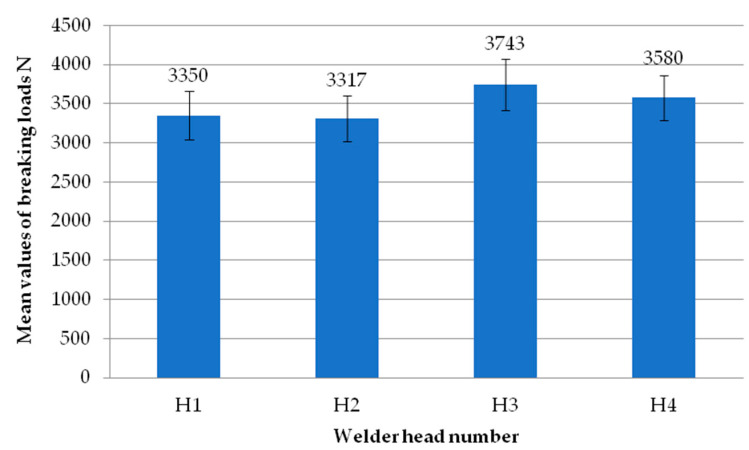
Average failure loads for individual heads using milling of the composite to a depth of 0.5 mm.

Figure 25 shows the average failure load values for each head. Each head represents the value of the average failure load from all the tests performed on that head using milling of the composite to a depth of 0.5 mm. There are no significant differences between the mean breaking load values for individual heads. The highest average value was for head three, while the lowest value was for head two. In contrast, all heads exceeded the break load value of 3300 N.

**Figure 26:**
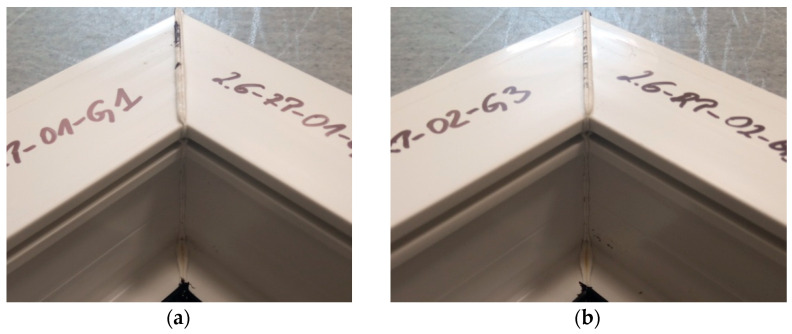
Two selected welds of test specimens using milling of the composite at a depth of 0.5 mm; (**a**)—for head one, (**b**)—for head three.

In Figure 26, welds of two different samples made using composite milling to a depth of 0.5 mm can be observed. All samples had a white flash colour (as expected). This indicates that the right welding temperature was selected—one that does not negatively affect the PVC profile through degradation. All samples maintained weld material continuity; i.e., in all samples, the occurrence of a weld seam could be observed. The symmetry of the welds was also tested. As a result, it was confirmed that there was no shifting of the profiles during the welding process.

### 3.8. Comparison of the Results of Performed Variants

**Figure 27:**
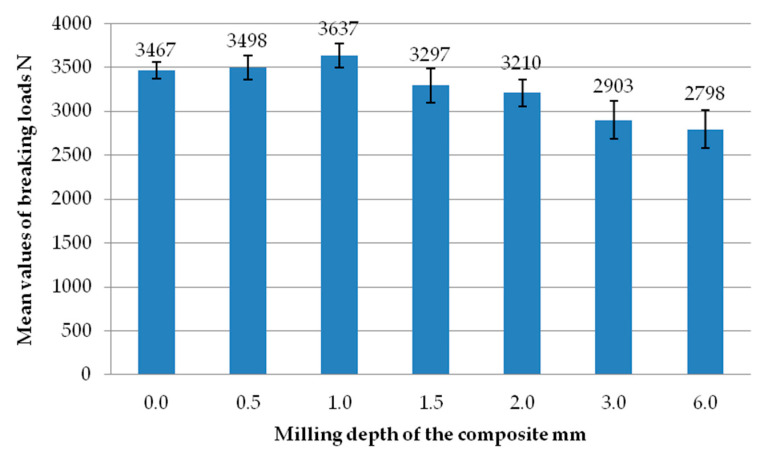
Average values of breaking loads of individual sets of samples with different degrees of composite milling.

Figure 27 presents a summary of the average destructive loads of individual samples divided by the depth of milling of the composite insert. The highest average destructive load is recorded by the set of samples concerning the milling of the composite with a value of 1 mm. The lowest average destructive load is recorded by the set of samples concerning the milling of the composite with a value of 6 mm. Among the set of samples concerning milling of the composite with values of 0 mm, 0.5 mm, and 1 mm, the highest average result concerns milling to a depth of 1 mm and an increasing trend of average values of destructive loads can be noted up to this milling depth. Above the milling depth of 1 mm, the trend is decreasing. Clear differences can be noted between the set of samples of the average value of destructive loads with the degree of milling of the composite of 1 mm and 1.5 mm. The set of samples with the degree of milling of 1.5 mm is characterised by a significantly lower average destructive load. In comparison to the subsequent sets of samples, this difference increased. In addition, it should be noted that in the case of the set of samples with a milling degree of 1.5 mm and deeper, a material discontinuity occurred in the weld area. The weld seam, which confirms the melting of the material and its bonding, was not visible. The reason was that the milling degree of the composite was too deep. There were no clear differences in the context of average breaking loads between the set of samples with a milling degree of 0 mm, 0.5 mm, and 1 mm.

## 4. Conclusions

Based on the conducted research, it can be concluded that the depth of machining of composite inserts significantly affects the quality of the welded joint in the PVC window frame. The quality of the joint was assumed to be not only the destructive force, but also the appearance of the flash and dimensional deviations.

It was found that the highest joint failure forces occurred in cases of milling to a depth of 0.5 mm, 1.0 mm and also without milling. On the other hand, the highest average value of the breaking load was recorded for a milling depth of the composite of 1 mm. This reached a value of 3637 N. In contrast, the average load values for milling the composite to a depth of 0.5 mm, and without milling the composite were, respectively, 3498 N and 3467 N. The mean values of the breaking loads for the other milling depth variants differed significantly in comparison to the mean value of the breaking load for milling to a depth of 1 mm. At the same time, it was noted that for this value, the flash was a visible binder covering the weld. The average value of the breaking load for a milling depth of the composite to a depth of 1.5 mm differs significantly from the set of samples with a degree of milling to a depth of 1 mm. Moreover, the occurrence of material discontinuities in this type of sample is disqualifying.

The conducted research confirmed that although the technology of connecting window frames has been known for many years, the use of modern materials (composite inserts) requires additional research in the area of production technology.

In future studies, the focus should be on broadening the spectrum within samples with a milling depth of up to 1 mm. Based on the studies conducted so far and their results, it is not possible to confirm with certainty which variant is the best. It is therefore necessary to conduct further research.

## Figures and Tables

**Table 1:** Characteristics of the PVC compound used for the profile [28].

Material	Vicat Softening Temperature °C	Notched Impact Strength kJ/m	Young’s Modulus of Elasticity N/mm	Linear Expansion Coefficient 1/K	Thermal Conductivity Coefficient W/m × K	Stability Time at 200 °C min
PVC-U	80–84	>40	2500–3000	7 × 10^−5^	0.16	40 ± 6

**Table 2:** Results of reconnaissance tests on the dimensional deviations of the welded PVC mould.

Sample No.	1	2	3	4	5	6
Dimensional deviations from nominal size in the X; Y axes [mm]	1; 1	0; 1	1; 0	1; 0	0; 1	0; 1

**Table 3:** Test results for milling the composite to a depth of 6.0 mm.

Sample No.	1	2	3
Dimensional deviations from nominal size in the X; Y axes [mm]	0; 0	−1; −1	0; 0

**Table 4:** Test results for milling the composite to a depth of 3.0 mm.

Sample No.	1	2	3
Dimensional deviations from nominal size in the X; Y axes [mm]	0; 0	−1; −1	−1; −1

**Table 5:** Test results for milling the composite to a depth of 2.0 mm.

Sample No.	1	2	3	4	5	6
Dimensional deviations from nominal size in the X; Y axes [mm]	0; 0	0; 0	0; 1	1; 1	0; 0	0; 1

**Table 6:** Test results for milling the composite to a depth of 1.5 mm.

Sample No.	1	2	3
Dimensional deviations from nominal size in the X; Y axes [mm]	1; 1	1; 1	0; 1

**Table 7:** Test results for milling the composite to a depth of 1 mm.

Sample No.	1	2	3	4	5	6
Dimensional deviations from nominal size in the X; Y axes [mm]	1; 1	1; 1	1; 1	0; 1	0; 0	0; 0

**Table 8:** Test results for milling the composite to a depth of 0.5 mm.

Sample No.	1	2	3
Dimensional deviations from nominal size in the X; Y axes [mm]	1; 1	1; 1	0; 1

## Data Availability

The original contributions presented in this study are included in the article. Further inquiries can be directed to the corresponding author.
